# Turning over New Leaves

**Published:** 1889-05-04

**Authors:** 


### Turning Over New Leaves.

(Books for Review should be sent to The Editor, The Lodge, Porchester Square, W.)

### SOME RECENT FICTION.*

It is not easy to give the reader a complete idea of this book in the column or so at our disposal; but we will try to present it to him as it appeared to us on a single perusal. First of all, the volume is well bound, tastefully covered, and neatly printed. There is a frontispiece, supposed to be taken from a painting, of which we can only echo the pious hope that the original was a better work of art than is the engraving.

The author is not altogether unknown to literature as a translator; but we do not remember to have seen any original work of his before, and it is our misfortune to be wholly unacquainted with the lady of many virtues to whom the book is inscribed. There is not much padding in the story, although forced incidents are, perhaps, a little too common, and the plot, such as it is, is easy enough to follow. It really consists of two biographies—that of uncle and nephew—and we are treated to an account of their sayings and doings in Europe, where the story, properly speaking, begins, and in America, where it ends. Richard Drysdale, the real hero of the book, is certainly an "unusual young man," and early in the volume we have a rather long description of his personal appearance, or "physique," as Mr. Sheldon would call it.

The uncle had early in life fallen in love with a professional singer whom he met in a railway carriage in France, married her, had a daughter who was brought up in Europe, and who, at the time the story opens, was about to join her father in the States, the father having meanwhile concealed everything about her from all his friends. What could be more natural than that the nephew should be invited to dine at the house of a rich, titled banker in London, there meet the young lady and fall in love with her without having the slightest notion she was his own cousin! The furniture in the baron's house is as carefully described as if the author were making out an auctioneer's list. "A grand Erard pianoforte, beside which stood a large, beautiful harp, caught the eye upon entering the multiple drawing-room, which evoluted spaciously." "Evoluted spaciously" is fine, but does it refer to the piano, the harp, or the multiple drawing-room? Even the livery of the servants does not escape the eye of the storyteller. Here is the introduction to the baron's house (or should we say mansion?) in Piccadilly: "They were ushered into the baronial hall by four periwigged flunkies liveried in white brocaded satin coats and knee breeches, wearing pink stockings gartered with rosettes below the knee, shod in enamelled pumps with silver buckles studded with brilliants, and gloved in spotless white kid." Being poor reviewers, anxious to pick up a guinea when our careful editor sends us a book to notice, we cannot be expected to have seen much of the inner life of the titled denizens of London; but we venture to express just the faintest shade of doubt whether flunkies often wear white brocaded satin coats, or have their shoes studded with diamonds. Anyway we do hope our Republican cousins will not think the practices are common among us. Then surely a baron's daughters would not have the courtesy titles of "Lady" Helena or "Lady" Fanny, and if they had it would sound a little strange to hear a father so describing his daughters to his guests in his own drawing-room; but a man who writes novels for us, and depicts the manners and customs of the great, ought to know better than we. The baron gave Richard Drysdale and his friends a good dinner, and, strange to say, a good supper, too.

The dialogue is often good, sometimes even sparkling; albeit many of the expressions jar a little on our ears now and then. We read "costumed" instead of "dressed"; "generous paper basket"; "opulent mansion"; "quite to the contrary"; "take Edith and I home." None of these are good English; some are not grammar.

The elder hero, Herbert Severance himself, says that when he went to France, he "frenchified his over-grammatical French," and the author has evidently followed his hero's example. It is astonishing how many of his French quotations are wrong. We notice, "Je n'ai sais quoi," "N'est pas," "Blazé," "Pas de etiquette," etc. Most astonishing, certainly, when we remember that the author actually translated a French story into English.

Clarisse, the heroine, writes a letter to her admirer, beginning, "Mr. Drysdale, my dear friend." The letter contains a liberal use of what the printers call "small caps; there are not fewer than eight French phrases, and we have, too, the same searching after "tall" English as in the book itself. Clearly, if Clarisse ever writes a novel, she will reproduce all Mr. Sheldon's faults. Nevertheless, we think he has considerable power of invention and a good deal of descriptive talent, and with care and study he is capable of writing a much better book than his maiden effort. Should he give us another book for review, we venture to hope he will avoid using French words and phrases where English ones are equally expressive, and that he will not ask his little heroine to perjure herself in a coroner's court, nor outvie Shelley by making her induce her father to swear he will kill a man who had wronged him.

* "Herbert Severance": a Novel. By M. French-Sheldon. Saxon and Co. Pp. 382.

### THE CHARACTERISTICS OF GENIUS.

Every contribution to the literature concerning mental philosophy is valuable, and must be welcomed in an age when mental acquirements and force are in such general demand. Dr. Gibson, in his essay, claims that "genius is an innate faculty of great minds, and holds its way through the activities of human life uncontrolled by passion and unsubdued by conventionalities." He builds up a strong case in favour of the theory that genius

"Must be born, and never can be taught.'

It is refreshing to peruse a work,* even so unpretentious as that before us, which does not support the clap-trap contentions of certain latter-day writers who flounder through a series of boisterous and unproved assertions, to the effect that genius is practically the same as industry. The chance remark of a somewhat able thinker, that this faculty "is an infinite capacity for taking pains," has gathered numerous adherents, probably because it is natural that one and all should wish to be endowed with genius. The theory that the genius is born and not made is a hard one, and its adoption could hardly be expected to take place so readily as that of the far pleasanter belief that all may be geniuses who are sufficiently industrious. But a study of the world's history, the history of individuals, of the arts, sciences, and of literature, leaves small doubt that certain individuals have been endowed with special abilities, which have enabled them to rise above the level of the great mass of humanity which has lived contemporarily with them.

Dr. Gibson conscientiously presents both sides of the question, commencing by enumerating those who have believed geniuses to be "little more than vigorous intellectual work governed by good common-sense." These form a goodly array, Helvetius, Hogarth, Newton, Descartes, and Mrs. Siddons being especially instanced. But it

* "The Characteristics of Genius." By Charles Gibson, M.D. Walter Scott, London. 1889.

would seem that the evidence of geniuses cannot always be taken as incontrovertible, and, acting on this assumption, the author proceeds, boldly enough, to state his case. "The man of genius has pre-eminence by an inborn power," he declares, "a power which is not simply the outcome of intellectual efforts," but which is "initiative, and which is innate, and which may be, and generally is, enhanced by education, by use, but which, nevertheless, lives and moves within, whether it is highly educated or not." Dr. Gibson divides genius into two forms—namely, the creative and constructive. As an example of the latter is quoted the case of Praxiteles, the celebrated sculptor of over 2,000 years ago, who, when invited to produce a figure of Venus, combined all the items of beauty in all his models, and thus constituted a whole which was ideally perfect. Homer, too, when forming the idea of Chimera, joined into one animal parts which belonged to several—the head of a lion, the body of a goat, and the tail of a serpent. The philosophers and poets are quoted as the creators who discover great fundamental truths in the sciences and arts, and who evolve forms of beauty to which our heightened and purified sense looks as ideals of perfection.

"The man of mere judgment," says the author, "is, indeed, essentially different from a man of genius. The former can exercise his reason only on subjects provided by others; the latter can provide subjects for himself." Milton and Dante are undoubted examples of men who possess extraordinary innate faculties. Coleridge was a poet and metaphysician at the age of fifteen, and Goethe displayed his genius before reaching his eighth year. Mozart was actually a composer at the age of four, whilst Locke, Plato, Euclid, and a host of others towered above their fellows in grand supremacy.

The eccentricities of genius are also dealt with by Dr. Gibson, who admits that many great mind have had peculiarities which more than justify the contention that genius is "closely allied to madness." But he reserves his opinion as to whether "an entirely novel view of a subject is the conception of a far-seeing genius, or the vision of a diseased brain." True it is that many eminent men have had the strangest proclivities. Richelieu, for example, found amusement in prancing round his billiard-table like a horse, kicking out, from time to time, at his servants as he passed. Goldsmith was "in wit a man; in simplicity a child." Francis Bacon was "the wisest, brightest, meanest of mankind." Peter the Great of Russia, after achieving a huge life's labour, abdicated in favour of his wife, walking on foot at the ceremony of her inauguration as captain of her guard. Ptolemy and Spinoza were eccentric; Tasso systematically feigned madness; Swift became passionless and idiotic; Chatterton, the boy poet, ended his short but marvellous career at eighteen; Shelley suffered the most extraordinary mental illusions and dejection; Cowper, whose prospects were the most brilliant, became insane; Julius Cæsar and the great Napoleon Buonaparte were epileptics; Newton, in middle life, suffered for a short period from mental aberration. The only drawback to the essay is that it is by far too short.

